# Retraction: Exogenous t-PA Administration Increases Hippocampal Mature BDNF Levels. Plasmin- or NMDA-Dependent Mechanism?

**DOI:** 10.1371/journal.pone.0255763

**Published:** 2021-08-03

**Authors:** The PLOS ONE Editors

After this article [1] was published, concerns were raised about the reported results.

Specifically:

- Similarities were noted between western blot bands reported in Figs 2, 3, 5, and 6. These concerns involve most of the western blot image data shown.
- There appear to be differences in band sequences across panels (i.e. similar bands abut dissimilar bands in different panels), and when image levels are adjusted there appear to vertical discontinuities between some lanes of Figs 3A, 3B, 5A, and 6. These issues suggest that images may have been spliced in assembling the figures.

The PLOS Publication Ethics team investigated these issues and asked the authors to comment on these concerns. The authors did not respond to our inquiries. However, after the retraction decision was communicated, the corresponding author acknowledged that identical western blot bands were used to represent protein expression in the article’s figures, but noted that the blot images were presented only as representative of the results and that the statistical analyses reflect analysis of data from three separate experiments. The authors did not provide original image data needed to support the results or clarify the image data reporting concerns, stating they were unable to do so due to the passage of time.

The concerns about the western blot data remain unresolved and call into question the reliability of the reported results. Therefore, the *PLOS ONE* Editors retract this article. We regret that the issues with the article’s figures were not identified prior to publication.

MR, APT, YB, CM, PG did not agree with retraction and stand by the article’s findings. The other authors either could not be reached or did not respond directly.
